# Extracorporeal membrane oxygenation for COVID-19: a systematic review and meta-analysis

**DOI:** 10.1186/s13054-021-03634-1

**Published:** 2021-06-14

**Authors:** Kollengode Ramanathan, Kiran Shekar, Ryan Ruiyang Ling, Ryan P. Barbaro, Suei Nee Wong, Chuen Seng Tan, Bram Rochwerg, Shannon M. Fernando, Shinhiro Takeda, Graeme MacLaren, Eddy Fan, Daniel Brodie

**Affiliations:** 1 Yong Loo Lin School of Medicine, National University of Singapore, Singapore, Singapore; 2 Cardiothoracic Intensive Care Unit, National University Heart Centre, National University Hospital, Singapore, 119228 Singapore; 3 Adult Intensive Care Services, Prince Charles Hospital, Brisbane, QLD Australia; 4 Queensland University of Technology, Brisbane, Australia; 5 University of Queensland, Brisbane, Australia; 6 Bond University, Gold Coast, QLD Australia; 7 Division of Paediatric Critical Care Medicine, University of Michigan, Ann Arbor, USA; 8 Child Health Evaluation and Research Center, University of Michigan, Ann Arbor, MI USA; 9 Saw Swee Hock School of Public Health, National University of Singapore, Singapore, Singapore; 10 Department of Medicine, Division of Critical Care, McMaster University, Hamilton, ON Canada; 11 Department of Health Research Methods, Evidence and Impact, McMaster University, Hamilton, ON Canada; 12 Division of Critical Care, Department of Medicine, University of Ottawa, Ottawa, ON Canada; 13 Japan ECMOnet for COVID-19 & President, Kawaguchi Cardiovascular and Respiratory Hospital, Saitama, Japan; 14 Interdepartmental Division of Critical Care Medicine, University of Toronto, Toronto, Canada; 15 Department of Medicine, Columbia University College of Physicians and Surgeons, New York, NY USA; 16 Center for Acute Respiratory Failure, New York-Presbyterian Hospital, New York, NY USA

**Keywords:** Extracorporeal membrane oxygenation, Acute respiratory distress syndrome, COVID-19, SARS-CoV-2

## Abstract

**Background:**

There are several reports of extracorporeal membrane oxygenation (ECMO) use in patients with coronavirus disease 2019 (COVID-19) who develop severe acute respiratory distress syndrome (ARDS). We conducted a systematic review and meta-analysis to guide clinical decision-making and future research.

**Methods:**

We searched MEDLINE, Embase, Cochrane and Scopus databases from 1 December 2019 to 10 January 2021 for observational studies or randomised clinical trials examining ECMO in adults with COVID-19 ARDS. We performed random-effects meta-analyses and meta-regression, assessed risk of bias using the Joanna Briggs Institute checklist and rated the certainty of evidence using the GRADE approach. Survival outcomes were presented as pooled proportions while continuous outcomes were presented as pooled means, both with corresponding 95% confidence intervals [CIs]. The primary outcome was in-hospital mortality. Secondary outcomes were duration of ECMO therapy and mechanical ventilation, weaning rate from ECMO and complications during ECMO.

**Results:**

We included twenty-two observational studies with 1896 patients in the meta-analysis. Venovenous ECMO was the predominant mode used (98.6%). The pooled in-hospital mortality in COVID-19 patients (22 studies, 1896 patients) supported with ECMO was 37.1% (95% CI 32.3–42.0%, high certainty). Pooled mortality in the venovenous ECMO group was 35.7% (95% CI 30.7–40.7%, high certainty). Meta-regression found that age and ECMO duration were associated with increased mortality. Duration of ECMO support (18 studies, 1844 patients) was 15.1 days (95% CI 13.4–18.7). Weaning from ECMO (17 studies, 1412 patients) was accomplished in 67.6% (95% CI 50.5–82.7%) of patients. There were a total of 1583 ECMO complications reported (18 studies, 1721 patients) and renal complications were the most common.

**Conclusion:**

The majority of patients received venovenous ECMO support for COVID-19-related ARDS. In-hospital mortality in patients receiving ECMO support for COVID-19 was 37.1% during the first year of the pandemic, similar to those with non-COVID-19-related ARDS. Increasing age was a risk factor for death. Venovenous ECMO appears to be an effective intervention in selected patients with COVID-19-related ARDS.

PROSPERO CRD42020192627.

**Supplementary Information:**

The online version contains supplementary material available at 10.1186/s13054-021-03634-1.

## Introduction

The use of extracorporeal membrane oxygenation (ECMO) for acute respiratory distress syndrome (ARDS) during outbreaks of emerging infections was previously reported during the 2009 influenza A(H1N1) pandemic, as well as the Middle East respiratory syndrome coronavirus (MERS-CoV) outbreaks [1–4]. More recently, there are reports on the use of ECMO in patients with coronavirus disease 2019 (COVID-19), who develop severe ARDS [5, 6]. The Extracorporeal Life Support Organisation (ELSO), the World Health Organization and the Surviving Sepsis Campaign (SSC) Guidelines recommend considering ECMO, in specialised centres, for patients with COVID-19 who develop severe ARDS. In addition, venovenous (VV) ECMO is recommended in selected patients who develop hypoxaemia that is refractory to optimal ventilator management and prone positioning, depending on the availability of resources [7–11].

Initial case reports and case series on the use of ECMO for COVID-19-related ARDS were disappointing and raised concerns regarding ECMO use in this patient population [12, 13]. However, several reports of ECMO use have subsequently emerged and have reported considerably better outcomes [5, 6, 14, 15]. The first update of SSC guidelines published recently, suggested that ECMO should be considered only for carefully selected patients with COVID-19 and severe ARDS, with a weak strength of recommendation [16]. Given the resource implications of providing ECMO in a pandemic and variability in the reported outcomes, we performed a systematic review of the literature to summarise outcome data during the first year of the pandemic and identify risk factors for an unfavourable outcomes in order to guide clinical decision-making and further research.

## Methods

### Search strategy and selection criteria

This study was registered with PROSPERO (CRD42020192627) and was conducted in adherence with the Preferred Reporting Items for Systematic Reviews and Meta-analyses (PRISMA) Statement [17]. We searched MEDLINE, Embase, Cochrane and Scopus databases from 1 December 2019, to 10 January 2021, using the following keywords and their variations: “extracorporeal membrane oxygenation”, “extracorporeal life support”, “adult”, “SARS-CoV-2” and “COVID-19” (Additional file 1: Table S1). We assessed all relevant studies and their citation lists to identify articles for inclusion.

We included data from all studies as well as available online national registries reporting on 10 or more adult patients with COVID-19 supported on ECMO. We excluded any animal or paediatric studies (< 18 years). In the case of overlapping patient data, we included the largest study and excluded any other overlapping studies. Studies from centres that contributed to the ELSO registry report [5] were also excluded to avoid duplication. Two reviewers (RRL and KR) independently screened the articles for eligibility by going through the titles and abstract. Full text of the shortlisted articles was searched thereafter; any conflicts were resolved by consensus or by a third reviewer (KS).

### Data collection

Data were collected independently by two reviewers (RRL and KR) using a prespecified data extraction form; any conflicts were resolved by consensus or by a third reviewer (KS). Data collection covered study characteristics (study design, study duration, year of publication, name and country of origin of ECMO centre, indications for ECMO); patient demographics (number of patients, proportion of male/female patients, age, body mass index [BMI], comorbidities); pre-ECMO characteristics (ventilation parameters: partial pressure of arterial oxygen to fraction of inspired oxygen ratio [PaO_2_/FiO_2_], serum pH, lactate, duration of mechanical ventilation before ECMO initiation, adjunctive therapies, Sequential Organ Failure Assessment [SOFA] score); ECMO characteristics (type of ECMO at initiation, cannulation site, adjunctive therapies [prone positioning, neuromuscular blockade, inotropes/vasopressors, inhaled nitric oxide, corticosteroids and immunomodulatory agents]); mortality (in-hospital as well as substitution of the closest common mortality time point); and other relevant clinical outcomes (intensive care unit [ICU] and hospital length of stay [LOS], ECMO duration and complications during ECMO). Complications were represented broadly as per the ELSO reporting guidelines. Authors were contacted for additional data where necessary.

### Assessment of risk of bias and certainty of evidence

Using the Joanna Briggs Institute (JBI) checklists for case series and cohort studies (Additional file 1: Table S2), we assessed studies for quality. We assessed the possibility of publication bias using Egger’s test. We assessed statistical heterogeneity using the *I*^2^ statistics, the Chi-squared test and visual inspection of the forest plots. We used the Grading of Recommendations, Assessments, Developments and Evaluations (GRADE) approach to assess the certainty of evidence[18, 19] (GRADEpro app available online: https://www.gradepro.org [accessed on 10 January 2021].

### Outcomes of interest

The primary outcome was in-hospital mortality. Secondary outcomes were analysed in the overall cohort and included those remaining in hospital and on ECMO, ICU and hospital length of stay, duration of mechanical ventilation before ECMO, duration of ECMO and complications during ECMO.

### Statistical analysis

We performed statistical analyses in R 3.6.1, using the *meta (v4.12-0) and dmetar (v0.0.9000)* packages [20–22]. For continuous variables, we pooled the means from the aggregate data presented in each study as per Wan et al. [23]. We anticipated significant interstudy heterogeneity given the varied presentation of COVID-19 and general lack of guidelines for patient management with ECMO during the early pandemic. As such, we conducted inverse-variance weighted random-effects meta-analyses (DerSimonian and Laird), and 95% confidence intervals (CIs) were computed using the Clopper–Pearson method [24–26]. Survival outcomes are presented as pooled proportions, while continuous outcomes are presented as pooled means, both with corresponding 95% CIs.

### Subgroup/sensitivity analysis

We conducted planned subgroup analyses with continuity correction to include studies with zero events and include geographical region (Asia, Europe, North America and International). Summary-level meta-regression was conducted when at least 6 data points were collected to explore potential sources of heterogeneity or prognostically relevant prespecified study-level covariates [26]. Two sensitivity analyses were conducted for our meta-analysis: analysing the mortality among studies where all patients were supported with VV ECMO and another by excluding studies with a JBI score of less than 7.

## Results

### Study details and demographics

**Fig. 1:**
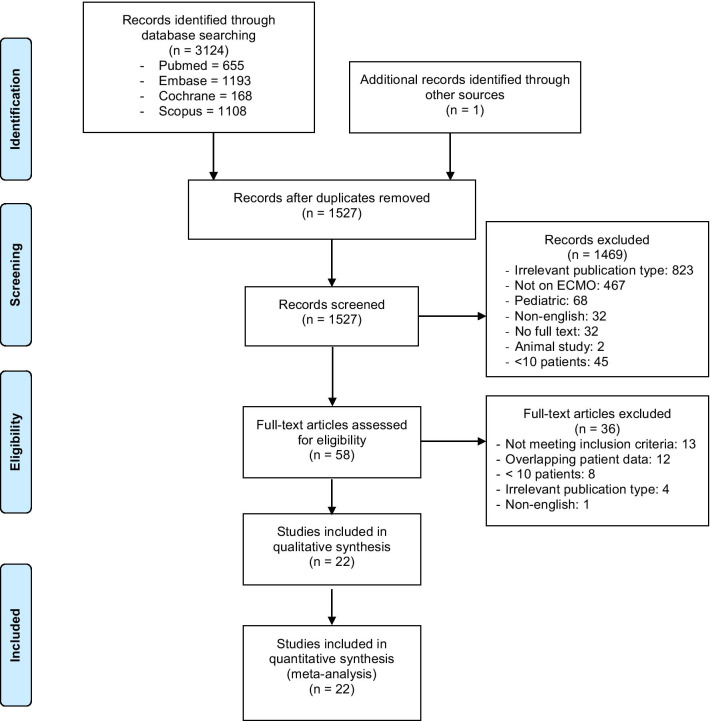
Preferred Reporting Items for Systematic Reviews and Meta-analyses (PRISMA) flow chart

Of 2259 references screened, we identified 37 potentially relevant studies and one national database that reported on the outcomes of ECMO in COVID-19 patients (Fig. 1) [5, 6, 12–15, 27–43]. After excluding 11 studies with overlapping information, we included twenty-two retrospective observational studies with 1896 patients in the meta-analysis. There were 20 single-centre studies and two registry reports (Tables 1 and 2). There were 4 studies (422 patients) from Asia, 13 studies (320 patients) from Europe, 4 studies (102 patients) from North America and one multinational study (1035 patients). Modality of ECMO support was reported in 19 studies (1845 patients), and VV ECMO was the predominant technique used (98.6%). In total, 89 patients required venoarterial or venoarterial venous configurations. The pooled patient demographics are summarised in Additional file 1: Table S3.

**Table 1:** Demographics of the included studies

First author	Country	Number of patients	Male patients n (%)	Age*	VV ECMO	P/F ratio*
Alnababteh	USA	13	8 (61.5)	44.54 ± 9.49	13	97.96 ± 49.87
Akhtar	UK	18	16 (88.9)	47.3 ± 0.9	18	NR
Barbaro	International	1035	764 (73.8)	49 (41–57)	978	72 (59–94)
Charlton	UK	34	27 (79.4)	46.3 ± 7.5	34	64.5 (54.7–74.3)
Cousin	France	30	24 (80)	33.33 ± 7.00	30	69 ± 9.34
Falcoz	France	17	16 (94.1)	56 [30–76]	16	71 [52–134]
Guihaire	France	24	20 (83.3)	48.8 ± 8.9	24	67 [52–78]
Huette	France	12	12 (100)	62 (58–64)	14	76 (66–83)
Jackel	Germany	15	11 (73.3)	60.8 (54.2–67)	15	63.7 (51.9–94.5)
Jang	Korea	19	15 (79)	63 ± 4.81	16	97.7 ± 61.11
Le Breton	France	13	10 (77)	49.31 ± 7.75	13	60.62 ± 15.23
Jozwiak	France	11	7 (63.6)	50 (38–59)	11	68 (58–89)
Masur	USA	12	8 (66.7)	53.83 ± 13.18	NR	NR
Mustafa	USA	40	30 (75)	48.4 ± 1.5	40	68.9 ± 3.1
Roedl	Germany	20	NR	NR	20	NR
Schmidt	France	83	61 (73.5)	48.0 ± 11.0	81	62 ± 18
Shih	USA	37	27 (73.0)	51 (40–59)	37	95 (73–147)
Takeda	Japan	237	196 (82.7)	NR	230	NR
Yang	China	21	12 (57.1)	58.5 (42.75–67.25)	21	60 (55.6–72)
Zayat	Germany	17	11 (64.7)	57 (53–62)	17	NR
Zeng	China	12	11 (91.7)	50.9 ± 13.5	NR	NR
Zhang	UK	43	33 (76.7)	46 (35.5–52.5)	43	67.5 (58.9–77.8)

VV ECMO, venovenous extracorporeal membrane oxygenation; P/F, partial pressure of arterial oxygen to fraction of inspired oxygen ratio [PaO2/FiO2]; NR, not reported

*Age and P/F ratio reported as mean ± SD, median (interquartile range) or median [range]

**Table 2:** Outcomes of the included studies

First author	Mortality	Survivors	Not discharged	Still on ECMO	Complications on ECMO	Days on ECMO*
Alnababteh	6	4	3	NR	3 mechanical 7 haemorrhagic 6 renal 4 pulmonary 2 infectious 3 metabolic 2 limb	12.85 ± 6.04
Akhtar	4	14	0	0		17.7 ± 9.4
Barbaro	380	588	67	31	295 mechanical 69 neurologic 444 renal	13.9 (7.8–23.3)
Charlton	16	18	0	0		
Cousin	16	NR	NR	NR	8 mechanical 27 haemorrhagic 4 neurologic 15 renal 2 pulmonary 4 infectious 3 limb	10.67 ± 5.45
Falcoz	6	10	1	0	7 mechanical 62 haemorrhagic 1 neurologic 12 renal 1 cardiovascular 3 pulmonary 10 infectious 1 others (thrombocytopenia)	9 [0–16]
Guihaire	4	16	4	3	18 mechanical 1 neurologic 10 pulmonary 3 infectious 1 other (mesenteric ischemia)	19.0 ± 10.1
Huette	4	8	0	0	5 mechanical 3 hemorrhagic 8 renal 1 cardiovascular 4 pulmonary 10 infectious 3 others (2 liver failure, 1 HIT)	12 (9–22)
Jackel	8	7	0	0		
Jang	10	4	5	2	5 neurologic 9 renal 1 cardiovascular 6 pulmomary	17.27 ± 16.42
Jozwiak	6	5	0	0		
Le Breton	2	11	0	0	2 mechanical 3 hemorrhagic 2 infectious	14.53 ± 8.84
Masur	5	1	6	NR	6 neurologic	9.60
Mustafa	6	29	5	2	10 pulmonary	29.9 ± 3.6
Roedl	13	7	0	0		
Schmidt	25	38	20	5	25 mechanical 45 hemorrhagic 5 neurologic 38 renal 11 cardiovascular 50 pulmonary 129 infectious 7 others (5 thrombocytopenia, 2 HIT)	20 (10–40)
Shih	16	21	0	0		
Takeda	67	NR	20	20	NA	14.42 ± 9.01
Yang	12	6	3	0	3 hemorrhagic 8 renal 8 cardiovascular 6 pulmonary 3 infectious	NR
Zayat	8	9	0	0		
Zeng	5	NR	7	4	NA	11.3 ± 7.8
Zhang	14	29	0	0		

ECMO, extracorporeal membrane oxygenation; NR, not reported

*Days on ECMO reported as mean ± SD, median (interquartile range) or median [range]

### Assessment of study quality

Appraisal using the JBI checklist for cohort studies and case series suggested a high level of quality across the included studies for this review. All studies, except two [13, 32], had scores above 8/10 (Additional file 1: Table S2). A summary of the GRADE assessment for certainty of evidence is provided in Additional file 1: Table S4.

### Pre-ECMO variables

Fifteen studies (1344 patients) reported on PaO_2_/FiO_2_ prior to ECMO initiation. The pooled mean PaO_2_/FiO_2_ was 67.76 (95% CI 64.72–70.80). Pre-ECMO SOFA score was reported in 11 studies (275 patients) with a pooled mean SOFA score prior to ECMO initiation of 9.62 (95% CI 8.40–10.84). The pre-ECMO ventilatory parameters are summarised in Additional file 1: Table S5.

### Pre-ECMO adjunctive therapies

Patients with COVID-19 who received ECMO also received various adjunctive therapies prior to ECMO. Pooled incidence of prone positioning prior to ECMO was 85.3% (95% CI 74.6–93.7%) while 96.3% (95% CI 87.6–100.0%) of the patients received neuromuscular blockers. Additional details on the use of inotropic agents, corticosteroids, immuno-modulators and antiviral drugs are highlighted in Additional file 1: Table S6**.**

### In-hospital mortality

**Fig. 2:**
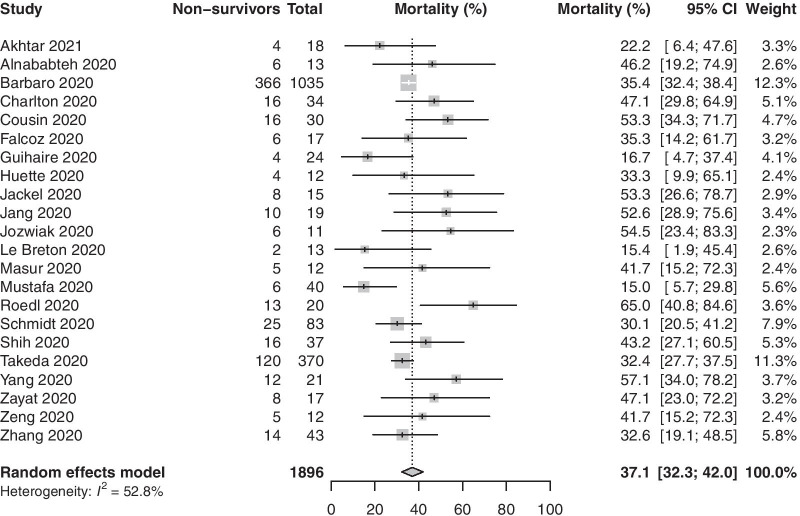
Proportion of non-survivors among coronavirus disease 2019 patients requiring extracorporeal membrane oxygenation support

**Fig. 3:**
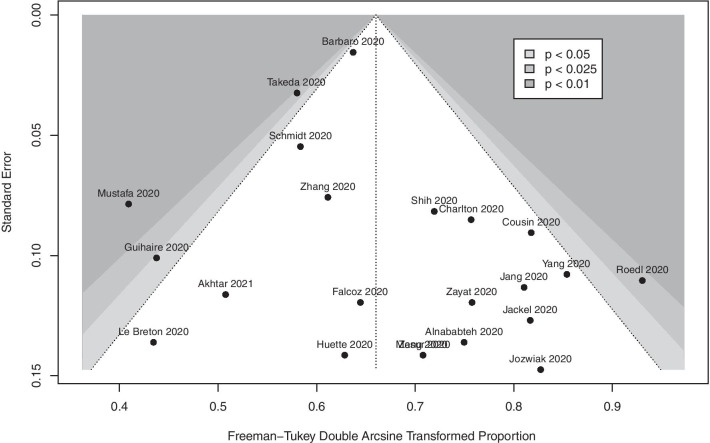
Funnel plot for primary meta-analysis

The pooled in-hospital mortality of COVID-19 patients receiving ECMO (22 studies, 1896 patients) was 37.1% (95% CI 32.3–42.0%, high certainty) (Fig. 2). Two studies had a JBI score lower than 8; pooled in-hospital mortality after excluding these studies was 37.9% (95% CI 32.9–42.9%). There was no evidence of publication bias (Fig. 3) (p_egger_ = 0.21). We also analysed the proportion of non-survivors supported on VV ECMO for COVID-19 (17 studies, 1737 patients); pooled in-hospital mortality was 35.7% (95% CI 30.7–40.7%, high certainty) (Additional file 2: Figure S1). Mortality, after removal of studies that did not report on pre-ECMO PaO_2_/FiO_2_ ratio, was 36.4% (95% CI 30.2–42.9%). Two studies compared mortality rates of patients on mechanical ventilation to those on ECMO; patients needing mechanical ventilation had mortality rates of 47.8% and 63.2% as compared to 46.15% and 57.1%, respectively, in those needing ECMO in these two studies.

### Subgroup analysis

There were no overall differences in regional outcomes for COVID-19 patients treated with ECMO (Additional file 3: Figure S2).

### Meta-regression analysis

Univariable meta-regression analysis identified increasing age and reduced ECMO duration as variables associated with mortality, while increasing BMI was protective (Additional file 4: Figure S3, Additional file 5: Figure S4 and Additional file 6: Figure S5). Increasing SOFA score was not associated with higher mortality. Other pre-ECMO factors (PaO_2_/FiO_2_ ratio, duration of mechanical ventilation prior to ECMO) or coexisting comorbidities (diabetes mellitus, hypertension, smoking) were not associated with increased mortality (Table 3).

**Table 3:** Results of meta-regression analyses

Covariate	Studies	OR	LCI	UCI	P
ECMO duration	19	0.987	0.979	0.994	**0.001**
Age	20	1.014	1.003	1.024	**0.01**
BMI	16	0.977	0.956	0.999	**0.04**
Male	21	0.566	0.314	1.017	0.06
Smoking	7	0.360	0.105	1.232	0.10
P/F ratio	15	1.003	0.998	1.007	0.25
Sample size	22	1.000	1.000	1.000	0.59
SOFA score	11	0.991	0.951	1.033	0.66
Ventilation-to-ECMO interval	16	0.997	0.945	1.051	0.90
DM	19	1.018	0.645	1.606	0.94
HTN	18	0.987	0.648	1.502	0.95

p-values: p < 0.05 represented in bold

OR, Odds ratio; LCI, lower 95% confidence interval; UCI, upper 95% confidence interval; P, p-value; ECMO, extracorporeal membrane oxygenation; SOFA, Sequential Organ Failure Assessment; BMI, body mass index; DM, diabetes mellitus; P/F, PaO2/FiO2; HTN, hypertension

### Secondary outcomes

In total, 141 of 1733 patients (21 studies) remained in hospital, while 68 of 1720 patients (20 studies) were still being supported with ECMO at the time of publication. Pooled ICU LOS (8 studies, 216 patients) and hospital LOS (6 studies, 1177 patients) were 32 days (95% CI 26–38, moderate certainty) and 40 days (95% CI 30–49, low certainty), respectively. There were 17 studies (1412 patients) that reported on weaning from ECMO with 67.6% (95% CI 50.5–82.7%, low certainty) of patients successfully weaned off ECMO. The pooled mean duration of mechanical ventilation prior to ECMO (16 studies, 1427 patients) was 4.40 days (95% CI 4.03–4.79, moderate certainty), while the pooled ECMO duration (18 studies, 1711 patients) was 15.81 days (95% CI 13.26–18.35, moderate certainty). Complications during ECMO were reported in 18 studies (1721 patients). There were a total of 1583 reported complications; renal complications (559/1583) were the most common, followed by mechanical (429/1583) and infectious complications (171/1583). A summary of all the outcomes including complications is provided in Table 2.

## Discussion

This systematic review and meta-analysis examined the use of ECMO in adult patients with COVID-19 during the first year of the pandemic. The pooled in-hospital mortality in 1896 patients with COVID-19 who predominantly had severe ARDS and were supported with ECMO was 37%, and this estimate was based on high certainty evidence. Mortality was slightly lower (35.7%) in patients exclusively receiving VV ECMO. This pooled mortality rate is comparable to the mortality rates seen in ECMO treated patients in the ECMO to Rescue Lung Injury in Severe ARDS [EOLIA] and Conventional ventilation or ECMO for Severe Adult Respiratory failure [CESAR] trials[44, 45] as well as the recent individual patient data meta-analysis of the two randomised controlled trials evaluating the use of VV ECMO in patients with ARDS from non-COVID etiologies (32.1 vs. 36%) [46]. The pooled mean duration of ECMO support in COVID-19 patients was 16 days, and the pooled mean ICU length of stay was 29 days. A large proportion of patients received neuromuscular blockade (96.2%) and were positioned prone (84.5%) prior to initiation of ECMO. The patients more commonly suffered renal, mechanical and infectious complications, and the complication rates in COVID-19 patients were similar to those seen in the EOLIA trial [44]. The review also identified increasing age as a risk factor for increased mortality.

The use and efficacy of ECMO in pandemics have been previously reported [1–3]. The incidence of ECMO use in patients with 2009 influenza A(H1N1)-associated ARDS in Australia and New Zealand was estimated to be 2–6 cases per million [1, 8], whereas 6% of critically ill patients were supported with ECMO for MERS-CoV-associated ARDS [4]. A meta-analysis of the studies that reported on the use of ECMO for the 2009 influenza A(H1N1) pandemic showed an overall mortality of 35% in a relatively younger population (mean age 40 years) [2]. Patients with MERS-CoV who were supported with ECMO had a higher reported mortality of 40–70% [3]. By comparison, this review demonstrated that the cumulative mortality for patients with COVID-19 receiving ECMO support was 37.1% in a group of patients who were older (mean age 51.6 years) and were predominantly men. These results may also alleviate some concerns regarding VV ECMO use in the context of COVID-19-related ARDS [13, 47]. During a pandemic, ECMO use will be subject to resource availability, given that prolonged ECMO support may be needed in these patients and the ELSO guidelines provide recommendations to assist clinicians in selecting patients judiciously in order to maximise benefit to patients with available resources [7, 8]. Our meta-regression analysis showed that outcomes appear worse in patients with increasing age. Clinicians may have to exercise considerable discretion when offering ECMO to older patients during a pandemic where resources may be stretched, with no definitive age cut-off to guide this decision-making.

Interestingly, greater duration of ECMO support and illness severity (SOFA score) were not independently associated with death. The duration of VV ECMO support was longer when compared with patients in the EOLIA trial receiving ECMO (median: 15.92 days vs 11 days). Prolonged ECMO runs outside COVID-19 have been reported with good success [48]. However, the association between prolonged ECMO duration and improved mortality seen in this study stems likely from immortal time bias[49](49), commonly reported in observational studies. For patients on ECMO, such individuals must survive long enough to be weaned off, whereas their peers have no minimum survival requirements. The mean duration of mechanical ventilation prior to ECMO was 4.4 days and was not associated with mortality in the meta-regression analysis. Higher SOFA score was not an independent predictor for death in this review, a finding that contradicts previously published experience [50]. SOFA scores in patients requiring ECMO for COVID-19 can be variable, depending on their underlying phenotype [51]. Therefore, caution should be exercised when placing patients with advanced extrapulmonary organ failures on ECMO. Even though VV ECMO appears to be a viable therapy, the potential need for prolonged ECMO support may be a significant consideration when selecting patients during the pandemic. Other uncertainties regarding the long-term outcomes and the maximum duration of ECMO where recovery is still possible remain and may become clearer as more data becomes available [52].

Strengths of this study include robust inclusion criteria and relevant exclusion criteria. Our review included 22 studies covering 6 geographical regions. We reduced confounding by elucidating factors correlating with mortality via subgroup analysis and meta-regression. Single-centre data that overlapped with international registries were excluded thereby avoiding duplication of data. We assessed study quality using a validated tool and assessed certainty in our estimates using GRADE. Nonetheless, we recognise several limitations of this study. Firstly, we included studies written only in English for the review. The variability in ECMO initiation and management across centres and regions as well as additional variability during the pandemic may have contributed to increased heterogeneity in our results. The outcomes of patients who were still in hospital or on ECMO at the time of publication were not known. Given that most of these studies were single-centre retrospective studies, these aspects could have introduced various confounders given the lack of risk adjustment or propensity score weighting. Even though the paper from ELSO registry contributed to a majority of patients in the review, the overall weightage to the entire analysis was only 12%, highlighting that the data were not skewed by one study. In addition, manuscripts published from centres that contributed to the ELSO registry report were excluded to avoid duplication. Meta-regression analyses are also inherently constrained by a lack of power, resulting in an increased risk of type 2 errors. This is further compounded by the fact that certain important variables, such as pre-ECMO SOFA scores, were only available in 11 studies, which might reduce the strength of the association between these variables and mortality Nonetheless, there was no publication bias in the studies included and JBI critical appraisal deemed most of the articles as high quality and suitable for inclusion while the GRADE assessment suggested a high certainty of evidence for the primary outcome.

## Conclusions

The outcomes from VV ECMO use in patients with COVID-19-related severe ARDS during the early pandemic appear similar to those reported in patients who receive VV ECMO for non-COVID-19-related severe ARDS. Increasing age is a risk factor for death. The duration of ECMO appears to be prolonged in patients with COVID-19 and a prolonged ECMO run was not in itself a predictor of death.

## Supplementary Information

**Additional file 1**. **Supplementary Tables:** Table S1 to S6.

**Additional file 2: Fig. S1**. Proportion of non-survivors among COVID-19 patients supported with venovenous ECMO.

**Additional file 3: Fig. S2**. Proportion of non-survivors among COVID-19 patients supported with ECMO stratified by geographical region.

**Additional file 4: Fig. S3**. Bubble plot correlating mean age and proportion of non-survivors.

**Additional file 5: Fig. S4**. Bubble plot correlating mean BMI and proportion of non-survivors.

**Additional file 6: Fig. S5**. Bubble plot correlating ECMO duration and proportion of non-survivors.

## Data Availability

All data generated or analysed during this study are included in the published studies and their supplementary information files.

## Abbreviations

ECMO: Extracorporeal membrane oxygenation
ARDS: Acute respiratory distress syndrome
MERS-CoV: Middle East respiratory syndrome coronavirus
COVID-19: Coronavirus disease 2019
ELSO: Extracorporeal Life Support Organisation
SSC: Surviving Sepsis Campaign
VV: Venovenous
PROSPERO: The International Prospective Register of Systematic Reviews
PRISMA: Preferred Reporting Items for Systematic Reviews and Meta-analyses
BMI: Body mass index
SOFA: Sequential Organ Failure Assessment
ICU: Intensive care unit
LOS: Length of stay
JBI: Joanna Briggs Institute
GRADE: Grading of Recommendations, Assessment, Development and Evaluations
EOILA: Extracorporeal membrane oxygenation for severe acute respiratory distress syndrome
CESAR: Conventional ventilatory support vs extracorporeal membrane oxygenation for severe adult respiratory failure
