# Correction to: Extracorporeal membrane oxygenation for COVID-19: a systematic review and meta-analysis

**DOI:** 10.1186/s13054-021-03714-2

**Published:** 2021-10-27

**Authors:** Kollengode Ramanathan, Kiran Shekar, Ryan Ruiyang Ling, Ryan P. Barbaro, Suei Nee Wong, Chuen Seng Tan, Bram Rochwerg, Shannon M. Fernando, Shinhiro Takeda, Graeme MacLaren, Eddy Fan, Daniel Brodie

**Affiliations:** 1 Yong Loo Lin School of Medicine, National University of Singapore, Singapore, Singapore; 2 Cardiothoracic Intensive Care Unit, National University Heart Centre, National University Hospital, Singapore, 119228 Singapore; 3 Adult Intensive Care Services, Prince Charles Hospital, Brisbane, QLD Australia; 4 Queensland University of Technology, Brisbane, Australia; 5 University of Queensland, Brisbane, Australia; 6 Bond University, Gold Coast, QLD Australia; 7 Division of Paediatrics Critical Care Medicine, University of Michigan, Ann Arbor, USA; 8 Child Health Evaluation and Research Center, University of Michigan, Ann Arbor, MI USA; 9 Saw Swee Hock School of Public Health, National University of Singapore, Singapore, Singapore; 10 Department of Medicine, Division of Critical Care, McMaster University, Hamilton, ON Canada; 11 Department of Health Research Methods, Evidence and Impact, McMaster University, Hamilton, ON Canada; 12 Division of Critical Care, Department of Medicine, University of Ottawa, Ottawa, ON Canada; 13 Japan ECMOnet for COVID-19 & President, Kawaguchi Cardiovascular and Respiratory Hospital, Saitama, Japan; 14 Interdepartmental Division of Critical Care Medicine, University of Toronto, Toronto, Canada; 15 Department of Medicine, Columbia University College of Physicians and Surgeons, New York, NY USA; 16 Center for Acute Respiratory Failure, New York-Presbyterian Hospital, New York, NY USA

## Correction to: Crit Care (2021) 25:211. 10.1186/s13054-021-03634-1

Following publication of the original article [1], the authors identified errors in Tables 1, 2, the Additional file 2: Figure S1 and Additional file 3: Figure S2. The correct Tables are is given hereafter and an explanation on the Additional file Figure S1 and Figure S2.

**Table 1:** Demographics of the included studies

First Author	Country	Number of patients	Male patients n(%)	Age*	VV ECMO	P/F ratio*
Alnababteh	United States of America	13	8 (61.5)	44.54 ± 9.49	13	97.96 ± 49.87
Akhtar	UK	18	16 (88.9)	47.3 ± 0.9	18	NR
Barbaro	International	1035	764 (73.8)	49 (41–57)	978	72 (59–94)
Charlton	UK	34	27 (79.4)	46.3 ± 7.5	34	64.5 (54.7–74.3)
Cousin	France	30	24 (80)	33.33 ± 7.00	30	69 ± 9.34
Falcoz	France	17	16 (94.1)	56 [30–76]	16	71 [52–134]
Guihaire	France	24	20 (83.3)	48.8 ± 8.9	24	67 [52–78]
Huette	France	12	12 (100)	62 (58–64)	14	76 (66–83)
Jackel	Germany	15	11 (73.3)	60.8 (54.2–67)	15	63.7 (51.9–94.5)
Jang	Korea	19	15 (79)	63 ± 4.81	16	97.7 ± 61.11
Le Breton	France	13	10 (77)	49.31 ± 7.75	13	60.62 ± 15.23
Jozwiak	France	11	7 (63.6)	50 (38–59)	11	68 (58–89)
Masur	United States of America	12	8 (66.7)	53.83 ± 13.18	NR	NR
Mustafa	United States of America	40	30 (75)	48.4 ± 1.5	40	68.9 ± 3.1
Roedl	Germany	20	NR	NR	20	NR
Schmidt	France	83	61 (73.5)	48.0 ± 11.0	81	62 ± 18
Shih	USA	37	27 (73.0)	51 (40–59)	37	95 (73–147)
Takeda	Japan	370	301(81.4)	NR	343	NR
Yang	China	21	12 (57.1)	58.5 (42.75–67.25)	21	60 (55.6–72)
Zayat	Germany	17	11 (64.7)	57 (53–62)	17	NR
Zeng	China	12	11 (91.7)	50.9 ± 13.5	NR	NR
Zhang	UK	43	33 (76.7)	46 (35.5–52.5)	43	67.5 (58.9–77.8)

VV-ECMO, venovenous extracorporeal membrane oxygenation; P/F, partial pressure of arterial oxygen to fraction of inspired oxygen ratio [PaO2/FiO2]; NR, not reported

^*^Age and P/F ratio reported as mean ± SD, median (interquartile range) or median [range]

**Table 2:** Outcomes of the included studies

First author	Mortality	Survivors	Not discharged	Still on ECMO	Complications on ECMO	Days on ECMO*
Alnababteh	6	4	3	NR	3 mechanical 7 haemorrhagic 6 renal 4 pulmonary 2 infectious 3 metabolic 2 limb	12.85 ± 6.04
Akhtar	4	14	0	0		17.7 ± 9.4
Barbaro	380	588	67	31	295 mechanical 69 neurologic 444 renal	13.9 (7.8–23.3)
Charlton	16	18	0	0		
Cousin	16	NR	NR	NR	8 mechanical 27 haemorrhagic 4 neurologic 15 renal 2 pulmonary 4 infectious 3 limb	10.67 ± 5.45
Falcoz	6	10	1	0	7 mechanical 62 haemorrhagic 1 neurologic 12 renal 1 cardiovascular 3 pulmonary 10 infectious 1 others (thrombocytopenia)	9 [0–16]
Guihaire	4	16	4	3	18 mechanical 1 neurologic 10 pulmonary 3 infectious 1 other (mesenteric ischemia)	19.0 ± 10.1
Huette	4	8	0	0	5 mechanical 3 hemorrhagic 8 renal 1 cardiovascular 4 pulmonary 10 infectious 3 others (2 liver failure, 1 HIT)	12 (9–22)
Jackel	8	7	0	0		
Jang	10	4	5	2	5 neurologic 9 renal 1 cardiovascular 6 pulmomary	17.27 ± 16.42
Jozwiak	6	5	0	0		
Le Breton	2	11	0	0	2 mechanical 3 hemorrhagic 2 infectious	14.53 ± 8.84
Masur	5	1	6	NR	6 neurologic	9.60
Mustafa	6	29	5	2	10 pulmonary	29.9 ± 3.6
Roedl	13	7	0	0		
Schmidt	25	38	20	5	25 mechanical 45 hemorrhagic 5 neurologic 38 renal 11 cardiovascular 50 pulmonary 129 infectious 7 others (5 thrombocytopenia, 2 HIT)	20 (10–40)
Shih	16	21	0	0		
Takeda	120	NR	NR	NR	NA	18.4 ± 15.2
Yang	12	6	3	0	3 hemorrhagic 8 renal 8 cardiovascular 6 pulmonary 3 infectious	NR
Zayat	8	9	0	0		
Zeng	5	NR	7	4	NA	11.3 ± 7.8
Zhang	14	29	0	0		

ECMO, extracorporeal membrane oxygenation; NR, not reported

*Days on ECMO reported as mean ± SD, median (interquartile range) or median [range]

Table 1 currently reads: Takeda et al. had 237 number of patients.

Table 1 should read: Takeda et al. had 370 patients ; 81.4% (301 patients) were males and 343 required VV ECMO. 

Table 2 currently reads Takeda et al. reported mortality in 67 patients.

Table 2 should read as Takeda et al. reported mortality in 120 patients with mean duration of 18.4 ± 15.2 days on ECMO.

The authors want to note that the forrest plot calculations in Fig. 2 was based on 370 patients. The article was written based on the forrest plot results (Fig. 2), which is the correct version.

The supplementary Figures 1 and 2 represent subgroup analysis based on 237 patients. However, on reanalysis, the final analysis results mostly remained the same, except for the confidence intervals, we have explained this in great detail in our rebuttal letter to Hoechter et al. [2]. The mortality on VV ECMO was 35.7% (95% CI: 30.8-40.7) while the regional mortality in Asia was 43.3% (95% CI: 28.9-58.3%).

All the changes that were requested are implemented in this correction and the original article [1] has been corrected.

## References

[1] Ramanathan K, Shekar K, Ling RR (2021). Extracorporeal membrane oxygenation for COVID-19: a systematic review and meta-analysis. Crit Care.

[2] Hoechter DJ, Becker-Pennrich AS, Geisler BP (2021). Letter to the editor regarding Extracorporeal membrane oxygenation for COVID-19: a systematic review and meta-analysis. Crit Care.

