# Supplementary material for: Extracorporeal membrane oxygenation for COVID-19: a systematic review and meta-analysis
Source: Crit Care. 2021 Jun 14;25:211. doi: 10.1186/s13054-021-03634-1 (PMC8201440; doi:10.1186/s13054-021-03634-1)
Supplement: Supplementary file 1 — Additional file 1. Supplementary Tables: Table S1 to S6. [file 13054_2021_3634_MOESM1_ESM.docx]

**Supplementary Material**

**Extracorporeal Membrane Oxygenation for COVID-19: A Systematic Review and Meta-Analysis**

Kollengode Ramanathan*, MD, FCICM^1,2^, Kiran Shekar*, FCICM, PhD^3,4^, Ryan Ruiyang Ling, MBBS^1^, Ryan P. Barbaro, MD, MSc^5^, Suei Nee Wong, MSc^1^, Chuen Seng Tan, PhD^1,6^, Bram Rochwerg, MD, MSc^7,8^, Shannon M. Fernando, MD, MSc^9^, Shinhiro Takeda, MD, PhD^10^, Graeme MacLaren, MSc^1,2^, FCICM, Eddy Fan, MD, PhD^11^, Daniel Brodie, MD^12,13^

***** Equally contributing authors

**Affiliations**

^1^ Yong Loo Lin School of Medicine, National University of Singapore, Singapore.

^2^ Cardiothoracic Intensive Care Unit, National University Heart Centre, National University Hospital, Singapore.

^3^ Adult Intensive Care Services and Critical Care Research Group, the Prince Charles Hospital, Brisbane, Queensland, Australia

^4^ Queensland University of Technology, Brisbane; University of Queensland, Brisbane and Bond University, Gold Coast, Queensland, Australia

^5^ Division of Paediatrics Critical Care Medicine, University of Michigan, Ann Arbor; Child Health Evaluation and Research Center, University of Michigan, Ann Arbor, Michigan, USA

^6^Department of Medicine, Division of Critical Care, McMaster University, Hamilton, ON, Canada

^7^ Saw Swee Hock School of Public Health, National University of Singapore, Singapore

^8^ Department of Health Research Methods, Evidence and Impact, McMaster University, Hamilton, ON, Canada

^9^ Division of Critical Care, Department of Medicine, University of Ottawa, Ottawa, ON, Canada

^10^ Chairman, Japan ECMOnet for COVID-19 & President, Kawaguchi Cardiovascular and Respiratory Hospital, Saitama, Japan

^11^ Interdepartmental Division of Critical Care Medicine, University of Toronto, Toronto, Canada

^12^ Department of Medicine, Columbia University College of Physicians and Surgeons, New York, NY, USA

^13^ Center for Acute Respiratory Failure, New York-Presbyterian Hospital, New York, NY, USA

Corresponding author:

Kollengode Ramanathan, Cardiothoracic Intensive Care Unit, National University Heart Centre, National University Hospital, Level 9, 1E Kent Ridge Road, SINGAPORE 119228.

Tel: +6567727862

Email: ram_ramanathan@nuhs.edu.sg.

ORCID : 0000-0003-1822-9455

**Supplementary Table 1. Search strategy for individual databases**

**Pubmed**

| 1. | Extracorporeal Membrane Oxygenation[MeSH] OR Extracorporeal Membrane Oxygenation[Title/Abstract] OR Extracorporeal Membrane Oxygenat*[Title/Abstract] OR Extracorporeal Life Support[Title/Abstract] OR ECMO[Title/Abstract] OR ECLS[Title/Abstract] OR Extracorporeal Circulat*[Title/Abstract] OR Extracorporeal[Title/Abstract] | 44,970 |
| --- | --- | --- |
| 2. | Severe acute respiratory syndrome coronavirus 2 [Supplementary Concept] OR COVID-19 [Supplementary Concept] OR ((2019[Title/Abstract] OR 19[Title/Abstract] OR wuhan[Title/Abstract] OR novel[Title/Abstract]) AND (COVID[Title/Abstract] OR coronavirus[Title/Abstract] OR nCoV[Title/Abstract] OR corona virus[Title/Abstract] OR CoV[Title/Abstract])) OR SARS-CoV-2[Title/Abstract] | 94,217 |
| 3. | #1 AND #2 | 655 |

**Embase**

| 1. | ‘Extracorporeal oxygenation’/exp OR ‘extracorporeal membrane oxygenation’:ti,ab OR ‘extracorporeal membrane oxygenat*’:ti,ab OR ‘extracorporeal life support’:ti,ab OR ‘ECMO’:ti,ab OR ‘ECLS’:ti,ab OR ’extracorporeal circulat*’:ti,ab OR extracorporeal:ti,ab | 71,237 |
| --- | --- | --- |
| 2. | ‘severe acute respiratory syndrome coronavirus 2’/exp OR ‘covid 19’/exp OR ‘coronavirus disease 2019’/exp OR ((2019 OR 19 OR wuhan) NEAR/10 (covid OR coronavirus OR nCoV OR “corona virus” OR CoV)):ti,ab OR SARSCoV-2:ti,ab | 94,470 |
| 3. | #1 AND #2 | 1193 |

**Cochrane**

| 1. | MeSH descriptor: [Extracorporeal Membrane Oxygenation] explode all trees | 178 |
| --- | --- | --- |
| 2. | (Extracorporeal Membrane Oxygenation OR Extracorporeal Membrane Oxygenat* OR Extracorporeal Life Support OR ECMO OR ECLS OR Extracorporeal Circulat* OR Extracorporeal):ti,ab | 4343 |
| 3. | #1 OR #2 | 4376 |
| 4 | (COVID-19 OR ((2019 OR 19 OR Wuhan OR novel) AND (COVID OR coronavirus OR nCoV OR corona virus OR CoV)) NEAR/10 SARS-CoV-2 OR severe acute respiratory syndrome coronavirus 2):ti,ab | 4039 |
| 5. | #3 AND #4 | 168 |

**Scopus**

| 1. | TITLE-ABS-KEY(“Extracorporeal Membrane Oxygenation” OR “Extracocporeal Membrane Oxygenat*” OR “Extracorporeal Life Support” OR “ECMO” OR “ECLS” OR “Extracorporeal Circulat*” OR “Extracorporeal”) | 75,584 |
| --- | --- | --- |
| 2. | TITLE-ABS-KEY(((“2019” OR “19” OR “Wuhan” OR “Novel”) W/10 (“COVID” OR “coronavirus” OR “nCoV” OR “corona virus” OR “CoV”)) OR “SARS-CoV-2” OR “severe acute respiratory coronavirus 2”) | 106,700 |
| 3. | #1 AND #2 | 1,108 |

**Supplementary Table 2. Joanna Briggs Institute checklist for case series and cohort studies**

| **Study** | **1** | **2** | **3** | **4** | **5** | **6** | **7** | **8** | **9** | **10** | **11** | **Score** |
| --- | --- | --- | --- | --- | --- | --- | --- | --- | --- | --- | --- | --- |
| Cohort studies | | | | | | | | | | | | |
| Alnababteh | **✓** | **✓** | **✓** | **✓** | **✗** | **✓** | **✓** | **✗** | **✓** | NA | **✓** | 8 |
| Barbaro | **✓** | **✗** | **✓** | **✓** | **✓** | **✓** | **✓** | **✓** | **✓** | **✓** | **✓** | 10 |
| Cousin | **✓** | **✓** | **✓** | **✓** | **✗** | **✓** | **✓** | **✓** | **✓** | NA | **✓** | 9 |
| Falcoz | **✓** | **✓** | **✓** | **✗** | **✗** | **✓** | **✓** | **✓** | **✓** | NA | **✓** | 8 |
| Guihaire | **✓** | **✓** | **✓** | **✗** | **✗** | **✓** | **✓** | **✗** | **✓** | NA | **✓** | 7 |
| Jang | **✓** | **✓** | **✓** | **✓** | **✓** | **✓** | **✓** | **✗** | **✓** | NA | **✓** | 9 |
| Mustafa | **✓** | **✓** | **✓** | **✗** | **✗** | **✓** | **✓** | **✓** | **✓** | NA | **✓** | 8 |
| Roedl | **✓** | **✓** | **✓** | **✓** | **✓** | **✓** | **✓** | **✓** | **✓** | NA | **✓** | 10 |
| Schmidt | **✓** | **✓** | **✓** | **✗** | **✗** | **✓** | **✓** | **✓** | **✓** | NA | **✓** | 8 |
| Takeda | NA, registry data not presented as a study, extracted directly from database | | | | | | | | | | | |
| Yang | **✓** | **✓** | **✓** | **✗** | **✓** | **✓** | **✓** | **✓** | **✓** | NA | **✓** | 9 |
| Case series | | | | | | | | | | | | |
| Akhtar | **✓** | **✓** | **✓** | **✓** | **✓** | **✓** | **✓** | **✓** | **✓** | **✓** |  | 10 |
| Charlton | **✓** | **✓** | **✓** | **✓** | **✓** | **✓** | **✓** | **✓** | **✓** | **✓** |  | 10 |
| Huette | **✓** | **✓** | **✓** | **✓** | **✓** | **✓** | **✓** | **✓** | **✓** | **✓** |  | 10 |
| Jozwiak | **✓** | **✓** | **✓** | **✓** | **✓** | **✓** | **✓** | **✓** | **✓** | **✓** |  | 10 |
| Le Breton | **✓** | **✗** | **✗** | **✓** | **✓** | **✓** | **✓** | **✓** | **✓** | **✓** |  | 8 |
| Masur | **✓** | **✓** | **✓** | **✗** | **✗** | **✓** | **✓** | **✓** | **✓** | **✓** |  | 8 |
| Shih | **✓** | **✓** | **✓** | **✓** | **✓** | **✓** | **✓** | **✓** | **✓** | **✓** |  | 10 |
| Zeng | **✗** | **✗** | **✗** | **✗** | **✗** | **✓** | **✓** | **✓** | **✓** | **✓** |  | 5 |

**Supplementary Table 3.** Pooled demographics of patients in included studies

| Demographic | Number of studies | Number of patients | Pooled result | 95% CI |
| --- | --- | --- | --- | --- |
| Age (years) | 19 | 1491 | 51.59 | 49.87-53.32 |
| Male patients | 21 | 1743 | 78.0% | 74.0%-81.7% |
| BMI | 16 | 1689 | 30.61 | 29.00-32.22 |

Abbreciations: CI: confidence interval, BMI: body mass index

**Supplementary Table 4: Grading of Recommendations, Assessments, Developments, and Evaluations (GRADE) Approach for assessing certainty of evidence**

| **№ of studies** | **Certainty assessment** | | | | | | **Effect** | | | **Certainty** | **Importance** |
| --- | --- | --- | --- | --- | --- | --- | --- | --- | --- | --- | --- |
|  | **Study design** | **Risk of bias** | **Inconsistency** | **Indirectness** | **Imprecision** | **Other considerations** | **№ of events** | **№ of individuals** | **Rate (95% CI)** |  |  |
| Pooled in-hospital mortality for all ECMO patients | | | | | | | | | | | |
| 22 | observational studies | not serious | not serious ^a^ | not serious | not serious ^b^ | none | - | 1763 | 37.0 %  (32.0 to 42.2) | ⨁⨁⨁⨁ HIGH | CRITICAL |
| Pooled in-hospital mortality for VV-ECMO patients | | | | | | | | | | | |
| 17 | observational studies | not serious | not serious ^c^ | not serious | not serious ^d^ | none | - | 1472 | 35.5 %  (30.3 to 40.8) | ⨁⨁⨁⨁ HIGH | CRITICAL |
| Intensive Care Unit Length of Stay (days) | | | | | | | | | | | |
| 8 | observational studies | not serious | not serious ^c^ | not serious | serious ^e,f^ | none | - | 216 | 32 days  (26 to 38) | ⨁⨁⨁◯ MODERATE | CRITICAL |
| Hospital length of stay (days) | | | | | | | | | | | |
| 6 | observational studies | not serious | serious ^g^ | not serious | serious ^e^ | none | - | 1177 | 40 days  (30 to 49) | ⨁⨁◯◯ LOW | CRITICAL |
| Successful liberation from ECMO | | | | | | | | | | | |
| 18 | observational studies | not serious | serious ^g^ | not serious | serious^e^ | none | - | 1686 | 67.9 %  (52.7 to 81.5) | ⨁⨁◯◯ LOW | CRITICAL |
| Duration of mechanical ventilation before ECMO (days) | | | | | | | | | | | |
| 16 | observational studies | not serious | serious ^g^ | not serious | not serious | none | - | 1427 | 4.40 days  (4.03 to 4.79) | ⨁⨁⨁◯ MODERATE | IMPORTANT |
| Duration of ECMO | | | | | | | | | | | |
| 18 | observational studies | not serious | not serious ^c^ | not serious | serious e | none | - | 1711 | 15.81 days  (13.26 to 18.35) | ⨁⨁⨁◯ MODERATE | IMPORTANT |

#### Explanations

a. There was some heterogeneity (I^2^ = 52%) in the point estimates. Nonetheless, the 95% CIs for the individual studies mostly overlapped with each other. Furthermore, meta-regression found that age, and ECMO duration were important contributors to this variability.

b. Overall, the sample size represents a considerable number of patients. While the 95% CI for the pooled estimate are relatively wide, the implications for ECMO initiation would be similar at both the upper and lower ends.

c. There was significant heterogeneity, and some variability in the point estimates in the forest plots. Nonetheless, most of the 95% CIs overlapped with each other. As such, although a borderline decision, we decided to not rate down for inconsistency.

d. The sample size represents a good number of patients. While the 95% CI is relatively wide, the implications for ECMO initiation would be similar at both the upper and lower ends.

e. The width of the 95% CI is wide, and holds important clinical and economic implications for patients. The decision to initiate ECMO might potentially change at both ends of the 95% CI. As such, we rated down for imprecision

f. Furthermore, the number of patients is small, below the optimal information size

g. There was important heterogeneity. Overall, the point estimates are sparsely distributed and the 95% CI only occasionally overlap.

Abbreviations: CI: confidence interval, ECMO: extracorporeal membrane oxygenation, VV: venovenou

**Supplementary Table 5:** Pre-ECMO ventilatory parameters

| Author | Tidal volume | Peak inspiratory pressure (cm H_2_O) | Plateau pressure (cm H_2_O) | Driving pressure (cm H_2_O) | Main airway pressure  (cm H_2_O) | Respiratory Rate (/min) | Positive end expiratory pressure (cm H_2_O) |
| --- | --- | --- | --- | --- | --- | --- | --- |
| Barbaro etal |  | 34 ± 6.7 |  |  |  | 25.33 ± 7.4234 | 14 ± 2.969 |
| Charlton etal | 476.3 ± 118.4 ml |  |  |  |  | 19.9 ± 4.8 | 13.3 ± 3.3 |
| Cousin etal | 6.4 ± 0.9 ml/kg |  |  | 16 ± 5.448 |  | 29.333 ± 4.671 | 14 ± 3.114 |
| Falcoz etal | 3.9 ± 1.2 ml/kg |  | 27.75 ± 5.2954 | 14.75 ± 4.1806 |  | 19 ± 4.459 | 12 ± 2.3 |
| Huette etal | 6.1 ± 0.2 ml/kg |  | 29 ± 1.6 |  | 31 ± 1.647 | 14 ± 1.647 |  |
| Jozwiak etal | 3.4 (2.5–3.6) ml/kg |  | 26 (25–28) | 8 (7–11) |  | 14 (12–16) | 18 (17-20) |
| Le Breton etal | 5.15 ± 0.73 ml/kg | - | 31.58 ± 0.79 | 20.08 ± 2.57 | 22.73 ± 2.24 |  |  |
| Mustafa etal | 429.4 ± 12.1 ml | 40.0 ± 2.0 | 32.7 ± 0.8 |  |  | 25.8 ± 1.1 | 17.0 ± 0.5 |
| Schmidt etal | 2.8 ± 1.8 ml/kg |  | 28 ± 2.263 | 12.7 ± 1.5 |  | 21.3 ± 3.0 | 12 (12-14) |
| Shih etal | 4 ml/kg | 10 | 24 (21.3-26) |  |  |  |  |
| Zayat etal |  | 28.6 (25.5-30.6) |  |  |  | 24.5 (22.4-30.6) | 14.3 (12.2-15.3) |

**Supplementary Table 6:** Adjunctive therapies

| Adjunctive therapies | Studies | Patients | Incidence of adjunctive therapy ( 95% CI) |
| --- | --- | --- | --- |
| Prone positioning | 15 | 1412 | 85.3% (95% CI: 74.6%-93.7%) |
| Neuromuscular blockade | 13 | 1379 | 96.3% (95% CI: 87.6%-100%) |
| Inotropes | 8 | 1235 | 57.9% (95% CI: 49.4%-66.2%) |
| Inhaled Nitric Oxide | 9 | 1231 | 55.4% (95% CI: 32.9%-76.8%) |
| Steroids | 10 | 1277 | 54.3% (95% CI: 37.3%-70.9%) |
| Lopinavir/Ritonavir | 5 | 1165 | 36.5% (95% CI: 12.1%-64.9%) |
| Hydroxychloroquine | 6 | 1128 | 37.4% (95% CI: 17.9%-59.2%) |
| Immunosuppressants | 4 | 1168 | 39.2% (95% CI: 17.5%-63.2%) |
| Remdesivir | 5 | 1209 | 17.8% (95% CI: 3.77%-37.8%) |

Abbreviations: CI: confidence interval

Figure captions:

**Supplementary Figure 1.** Proportion of nonsurvivors among COVID-19 patients supported with veno-venous ECMO.

**Supplementary Figure 2.** Proportion of nonsurvivors among COVID-19 patients supported with ECMO stratified by geographical region

**Supplementary Figure 3.** Bubble plot correlating mean age and proportion of non-survivors

**Supplementary Figure 4.** Bubble plot correlating mean BMI and proportion of non-survivors

**Supplementary Figure 5.** Bubble plot correlating proportion of male patients and non-survivors
